# Assumption-Free Derivation of the Bell-Type Criteria of Contextuality/Nonlocality

**DOI:** 10.3390/e23111543

**Published:** 2021-11-19

**Authors:** Ehtibar N. Dzhafarov

**Affiliations:** Department of Psychological Sciences, Purdue University, West Lafayette, IN 47907, USA; ehtibar@purdue.edu

**Keywords:** Bell criteria, contextuality, context-independent mapping, free choice, local causality, no-fine-tuning, nonlocality

## Abstract

Bell-type criteria of contextuality/nonlocality can be derived without any falsifiable assumptions, such as context-independent mapping (or local causality), free choice, or no-fine-tuning. This is achieved by deriving Bell-type criteria for inconsistently connected systems (i.e., those with disturbance/signaling), based on the generalized definition of contextuality in the contextuality-by-default approach, and then specializing these criteria to consistently connected systems.

## 1. Introduction

The criteria (necessary and sufficient conditions) of contextuality/nonlocality (usually, but not necessarily, in the form of inequalities) are at the heart of the foundations of quantum physics. Since John Bell’s pioneering work, researchers have been interested in what assumptions about nature one needs to justify these criteria. Several such assumptions have been proposed. One of them is that measurements cannot be affected by spacelike remote events (*local causality*). Another assumption is that experimenters choose what they measure independent of the background events determining measurement outcomes (*free choice*). It has also been proposed that statistically identical measurement outcomes should have identical ontological models (*no-fine-tuning*). This article shows that the criteria of contextuality/nonlocality can be derived without any such assumptions.

Although our discussion is valid for essentially all possible systems of random variables, we will use a system describing the Bohm’s version of the Einstein-Podolsky-Rosen experiment (EPR/B) [1,2,3] as a throughout example. This allows us to avoid technicalities needed in a more general exposition. The system in question is

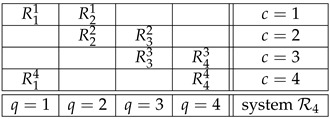
.
(1)

The random variables in (1) represent outcomes of spin measurements of two entangled particles along direction *q*; in every trial Alice chooses between directions q=1 and 3, and Bob chooses between q=2 and 4 (indicated by subscripts of the random variables). We call subscripts *q* in $R_{q}^{c}$ the *contents* of the corresponding random variables. The four combinations of the Alice-Bob choices form *contexts* c=1,…,4 (indicated by the superscripts of the random variables). If the spin along an axis *q* (i.e., content *q*) is measured in context *c*, we write:

(2) q≺c.

For instance q=2≺c=1 but q=3⊀c=1. The particles are spin-½, so all the random variables in the system are dichotomous, say ±1.

The structure of this article is as follows. In Section 2, we do preliminary work: we present a rigorous version of the traditional account of the criteria for contextuality/nonlocality of systems of random variables with no disturbance (we call them consistently connected systems), and then we stipulate the traditional assumptions involved in the formulation of these criteria. The main point of this paper is presented in Section 3. There, we generalize the definition of contextuality/nonlocality to inconsistently connected systems (those with disturbance or signaling), and show that the generalized Bell-type criteria in this generalized conceptual setting can be derived with no assumptions. Instead they are derived from a classificatory definition of (non)contextuality based on the notion of the difference between random variables. The traditional Bell-type criteria then immediately follow by specializing the generalized criteria to consistently connected systems, again, with no assumptions about nature.

The term “nonlocality” has been criticized as misleading, unnecessary, or even outright contradicting the tenets of quantum physics in several recent publications [4,5,6]. In this paper, however, the term is used to simply designate a special case of contextuality, for systems where contexts are formed by spacelike separated components. Barring experimental biases, such a system is consistently connected, and its mathematical analysis does not differ from that of other consistently connected systems [7,8]. Singling nonlocal systems out, however, is justified due to their special importance in quantum physics.

## 2. Contextuality for Consistently Connected Systems

Consider a random variable Λ and a measurable function *F* mapping Λ and *q* into $\left\{-1,1\right\}$. In contextuality/nonlocality analysis of a system we are asking whether one can choose $\left(\Lambda ,F\right)$ so that, for every context *c*,

(3) $$\left(F\left(q,\Lambda \right):q≺c\right)\overset{d}{=}\left(R_{q}^{c}:q≺c\right),$$

where $\overset{d}{=}$ stands for “has the same distribution as”. In other words, the joint distribution of all $F\left(q,\Lambda \right)$ for a given context *c* is the same as the joint distribution of all $R_{q}^{c}$ in this context. Thus, for system $R_{4}$ in (1),

(4) $$\begin{array}{c} \left(F\left(q=1,\Lambda \right),F\left(q=2,\Lambda \right)\right)\overset{d}{=}\left(R_{1}^{1},R_{2}^{1}\right),\\ \left(F\left(q=2,\Lambda \right),F\left(q=3,\Lambda \right)\right)\overset{d}{=}\left(R_{2}^{2},R_{3}^{2}\right),\\ etc. \end{array}$$

Note that the random variables $\left(R_{q}^{c}:q≺c\right)$ in a given context *c* are jointly distributed: e.g., the event $\left[R_{1}^{1}=x,R_{2}^{1}=y\right]$ is well-defined for any $x,y\in \left\{-1,1\right\}$, and has a probability assigned to it. At the same time, random variables in different contexts, e.g., $R_{1}^{1}$ and $R_{1}^{2}$, or $R_{1}^{1}$ and $R_{2}^{2}$, do not have a joint distribution, as different contexts are mutually exclusive [9]. However, all random variables $F\left(q,\Lambda \right)$ are jointly distributed, because Λ is one and the same for all *q* and *c*. Therefore, the random variables $S_{q}^{c}$ below form a (probabilisitic) *coupling* of system $R_{4}$:
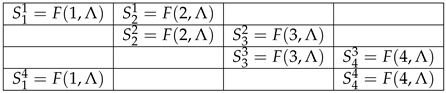
.
(5)

Generally, a coupling of a system of random variables $R=\left\{R_{q}^{c}:c\in C,q\in Q,q≺c\right\}$ is a set of jointly distributed $S=\left\{S_{q}^{c}:c\in C,q\in Q,q≺c\right\}$ such that, for any c∈C,

(6) $$S^{c}=\left\{S_{q}^{c}:q\in Q,q≺c\right\}\overset{d}{=}\left\{R_{q}^{c}:q\in Q,q≺c\right\}=R^{c}.$$

Note that any set of jointly distributed random variables is a random variable. Therefore, *S*, $S^{c}$, and $R^{c}$ are random variables, but R is not (hence the difference in notation).

In the coupling (5), the octuple of the $S_{q}^{c}$-variables is jointly distributed, and the within-context joint distributions of $S_{q}^{c}$ variables are the same as the joint distributions of the corresponding $R_{q}^{c}$ variables. In addition, since $F\left(q,\Lambda \right)$ does not depend on *c*, we have (with Pr standing for probability):

(7) $$\mathrm{Pr}\left[S_{q}^{c}=S_{q}^{c^{\prime }}\right]=1,$$

for any $q≺c,c^{\prime }.$ In other words, the probabilities

(8) $$\mathrm{Pr}\left[S_{1}^{1}=s_{1}^{1},S_{2}^{1}=s_{2}^{1},\ldots ,S_{4}^{4}=s_{4}^{4},S_{1}^{4}=s_{1}^{4}\right]$$

are well-defined for all $2^{8}$ octuples of $s_{q}^{c}\in \left\{-1,1\right\}$. These probabilities are subject to the following constraints:

1. For every fixed $\left(c,q,q^{\prime }\right)$, such that $q,q^{\prime }≺c$, and any fixed values $s_{q}^{c},s_{q^{\prime }}^{c}\in \left\{-1,1\right\}$,
  (9) $$\sum \iota_{\left[S_{q}^{c}=s_{q}^{c},S_{q^{\prime }}^{c}=s_{q^{\prime }}^{c}\right]}\mathrm{Pr}\left[S_{1}^{1}=s_{1}^{1},\ldots ,S_{1}^{4}=s_{1}^{4}\right]=\mathrm{Pr}\left[R_{q}^{c}=s_{q}^{c},R_{q^{\prime }}^{c}=s_{q^{\prime }}^{c}\right],$$
  where the coefficient $\iota_{\left[expression\right]}$ is the Boolean indicator of whether the event $S_{1}^{1}=s_{1}^{1},\ldots ,S_{1}^{4}=s_{1}^{4}$ contains expression;
2. For any fixed $\left(c,c^{\prime },q\right)$ such that $q≺c,c^{\prime }$, and any fixed values $s_{q}^{c},s_{q}^{c^{\prime }}\in \left\{-1,1\right\}$,
  (10) $$\sum \iota_{\left[S_{q}^{c}=s_{q}^{c},S_{q}^{c^{\prime }}=s_{q}^{c^{\prime }}\right]}\mathrm{Pr}\left[S_{1}^{1}=s_{1}^{1},\ldots ,S_{1}^{4}=s_{1}^{4}\right]=\left\{\begin{array}{ccc} 0 & if & s_{q}^{c}\neq s_{q}^{c^{\prime }}\\ \mathrm{Pr}\left[R_{q}^{c}=s_{q}^{c}\right]=\mathrm{Pr}\left[R_{q}^{c^{\prime }}=s_{q}^{c}\right] & if & s_{q}^{c}=s_{q}^{c^{\prime }} \end{array}\right..$$

This can be compactly presented as a linear programing (LP) task [7,10]:

(11) MX=P,X≥0,

where X is the vector of the $2^{8}$ probabilities (8), P is the vector of the probabilities in the right-hand sides of (9) and (10), and M is a Boolean incidence matrix, whose entries are the ι-coefficients in (9) and (10). The condition X≥0 (componentwise) ensures that the solution X, if it exists, consists of numbers interpretable as probabilities (the summation of these values to 1 in (11) is ensured).

Denoting by $\mathrm{LP}\left[M,P\right]$ a Boolean function that equals 1 if and only if a solution X exists, it is well-known that $\mathrm{LP}\left[M,P\right]$ is computable in polynomial time [11]. Consequently,

(12) $$\mathrm{LP}\left[M,P\right]=1$$

can be taken as a criterion of noncontextuality/locality. As an optional step, by a facet enumeration algorithm, this criterion can be presented in the form of inequalities and equations involving moments of the distributions within contexts. For system $R_{4}$, this yields [3,12,13]:

(13) $$\left|\langle R_{1}^{1}R_{2}^{1}\rangle +\langle R_{2}^{2}R_{3}^{2}\rangle +\langle R_{3}^{3}R_{4}^{3}\rangle +\langle R_{4}^{4}R_{1}^{4}\rangle -2\min\left(\langle R_{1}^{1}R_{2}^{1}\rangle ,\langle R_{2}^{2}R_{3}^{2}\rangle ,\langle R_{3}^{3}R_{4}^{3}\rangle ,\langle R_{4}^{4}R_{1}^{4}\rangle \right)\right|\leq 2,$$

(14) $$\langle R_{1}^{1}\rangle =\langle R_{1}^{4}\rangle ,\langle R_{2}^{1}\rangle =\langle R_{2}^{2}\rangle ,\langle R_{3}^{2}\rangle =\langle R_{3}^{3}\rangle ,\langle R_{4}^{3}\rangle =\langle R_{4}^{4}\rangle .$$

Equalities (14) present the condition of consistent connectedness.

What ontological assumptions have we made in the foregoing? Let us note first that we need no assumptions to derive the criterion $\mathrm{LP}\left[M,P\right]=1$ or its equivalents from (3). The derivation of the criterion is by straightforward linear programming, optionally complemented by facet enumeration. However, we need certain assumptions about nature to justify the plausibility of using the function $F\left(q,\Lambda \right)$, which is the starting point of the derivation. Namely, we must have assumed that the mapping does not contain context *c* among its arguments. For the EPR/Bohm experiment, following Bell [14], this assumption can be called *local causality*, because dependence of the mapping on *c* can be interpreted as dependence of measurement outcomes on spacelike-remote settings. More generally, however, this can be called *context-independent mapping*, in order to also include the Kochen-Specker type contextuality [15], when measurements in the same context are not spacelike separated. We must have also assumed that Λ in $F\left(q,\Lambda \right)$ is one and the same for all *q* and for all *c*. This is called the assumption of *free choice*, or *statistical independence*(of measurements and settings). In relation to system $R_{4}$, the necessity of adding the free choice assumption to the local causality assumption was pointed out to John Bell by Shimony, Horne, and Clauser in their 1985 interchange [16,17]. The relationship between free choice and local causality (more generally, context-independent mapping) is an interesting issue, but it is discussed elsewhere [18].

One can replace both the assumption of context-independent mapping and the free choice assumption with the assumption proposed by Cavalcanti, called *no-fine-tuning* [19,20]. For our purposes it can be formulated thus: if two random variables sharing a content in different contexts have the same distribution, their representations in the form

*F*(some parameters, some random variables) (15)

should be identical. This is an attractive alternative to context-independent mapping, because the latter is not especially compelling in the cases of Kochen-Specker-type contextuality, when contexts are not defined by spacelike remote settings. Note that the no-fine-tuning can also be considered a principle of theory construction, essentially a conceptual parsimony principle, rather than an ontological assumption.

As it turns out, however, by generalizing the notion of (non)contextuality to include inconsistently connected systems, one can avoid the necessity of making any of these, or other, falsifiable assumptions. The no-fine-tuning assumption (or parsimony principle) is a consequence of specializing this general definition to consistently connected systems.

## 3. Contextuality in Inconsistently Connected Systems

The generalization follows the broadening of the class of systems of random variables amenable to contextuality analysis. As one can see in (14), the criterion $\mathrm{LP}\left[M,P\right]=1$ can be satisfied only if the system of random variables is consistently connected: random variables measuring the same property in different contexts have the same distribution. We will now drop this constraint, and allow for inconsistent connectedness. In particular, we allow for signaling between Alice and Bob if their measurements are timelike separated.

We begin with the maximally lax representation

(16) $$R_{q}^{c}=\gamma \left(q,c,\lambda_{q}^{c}\right),$$

in which both the outcome of the measurement and the background random variable are allowed to depend on both *q* and *c*. This representation obviously holds for any system of random variables. Now, because all $R_{q}^{c}$ in a given context *c* are jointly distributed, it follows that all $\lambda_{q}^{c}$ in this context are jointly distributed. Therefore, there is a random variable $\lambda^{c}$ of which $\lambda_{q}^{c}$ for all q≺c are functions. As a result, we get a seemingly more restrictive but still universally applicable representation:

(17) $$R_{q}^{c}=g\left(q,c,\lambda^{c}\right).$$

The remaining dependence of $\lambda^{c}$ on context *c* can also be eliminated, by the following reasoning. Let us form an arbitrary coupling

(18) $$\Lambda \overset{d}{=}\left(\lambda^{c}:\mathrm{all}\;c\right),$$

e.g., couple all $\lambda^{c}$ independently. Then:

(19) $$R_{q}^{c}\overset{d}{=}g\left(q,c,{\mathrm{Proj}}_{c}\left(\Lambda \right)\right)\overset{}{=}G\left(q,c,\Lambda \right),$$

where ${\mathrm{Proj}}_{c}$ is the *c*th projection function. This representation (with Λ one and the same for all *q* and *c*) is also universally applicable. Note that in (19) we only have $R_{q}^{c}\overset{d}{=}G\left(q,c,\Lambda \right)$, rather than $R_{q}^{c}=G\left(q,c,\Lambda \right)$, because the latter would make $R_{q}^{c}$ jointly distributed across mutually exclusive contexts *c* (which is nonsensical).

It remains to define (non)contextuality in this generalized conceptual setting. In the contextuality-by-default approach (CbD) [8,9,10,21,22], the system is considered noncontextual if $\left(\Lambda ,G\right)$ in (19) can be chosen so that the probability:

(20) $$\mathrm{Pr}\left[G\left(q,c,\Lambda \right)=G\left(q,c^{\prime },\Lambda \right)\right]$$

is the maximal possible, for all $\left(q,c,c^{\prime }\right)$ such that $q≺c,c^{\prime }$. For dichotomous random variables, this means

(21) $$\mathrm{Pr}\left[G\left(q,c,\Lambda \right)=G\left(q,c^{\prime },\Lambda \right)=1\right]=\min\left\{\begin{array}{c} \mathrm{Pr}\left[R_{q}^{c}=1\right]\\ \mathrm{Pr}\left[R_{q}^{c^{\prime }}=1\right] \end{array}\right..$$

The rationale for this definition is as follows. The maximal probability with which $G\left(q,c,\Lambda \right)$ and $G\left(q,c^{\prime },\Lambda \right)$ can be made to coincide shows how similar the random variables $R_{q}^{c}$ and $R_{q}^{c^{\prime }}$ are if taken as an isolated pair, “out of their contexts”. Denoting by $p_{q}^{c}$ the value of $\mathrm{Pr}\left[F\left(q,c,\Lambda \right)=1\right]=\mathrm{Pr}\left[R_{q}^{c}=1\right]$, the maximum probability of $F\left(q,c,\Lambda \right)=F\left(q,c^{\prime },\Lambda \right)$ is $1-\left|p_{q}^{c}-p_{q}^{c^{\prime }}\right|$, and it is achieved if and only if the joint distribution of $S_{q}^{c}=F\left(q,c,\Lambda \right)$ and $S_{q}^{c^{\prime }}=F\left(q,c^{\prime },\Lambda \right)$ is (assuming $p_{q}^{c}\leq p_{q}^{c^{\prime }}$):
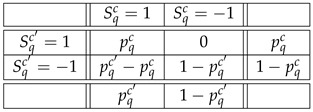
.
(22)

The existence of a coupling of the system in which all pairs $\left(S_{q}^{c},S_{q}^{c^{\prime }}\right)$ have this joint distribution indicates that the way $R_{q}^{c}$ and $R_{q}^{c^{\prime }}$ are related to other random variables in the corresponding contexts leaves their dissimilarity intact. Otherwise, the contexts of the system “force” some of the pairs of $R_{q}^{c}$ and $R_{q}^{c^{\prime }}$ to be more dissimilar than they are when taken in isolation.

The Bell-type criterion for this generalized definition is also determined by the LP problem (11). The only difference is that the part of vector P determined by (10) is replaced with the probabilities given in (22):

(23) $$\sum \iota_{\left[S_{q}^{c}=s_{q}^{c},S_{q}^{c^{\prime }}=s_{q}^{c^{\prime }}\right]}\mathrm{Pr}\left[S_{1}^{1}=s_{1}^{1},\ldots ,S_{1}^{4}=s_{1}^{4}\right]=\left\{\begin{array}{ccc} p_{q}^{c} & if & s_{q}^{c}=s_{q}^{c^{\prime }}=1\\ 1-p_{q}^{c^{\prime }} & if & s_{q}^{c}=s_{q}^{c^{\prime }}=-1\\ \; & etc. \end{array}\right..$$

Note that this definition of (non)contextuality is purely classificatory, it does not involve any assumptions about nature.

Suppose now that the system is consistently connected. Then the maximal probability in (20) is 1, i.e., (22) holds with $p_{q}^{c}=p_{q}^{c^{\prime }}$, and the system is noncontextual if and only if $\left(\Lambda ,G\right)$ can be chosen so that:

(24) $$\mathrm{Pr}\left[G\left(q,c,\Lambda \right)=G\left(q,c^{\prime },\Lambda \right)\right]=1,$$

whenever $q≺c,c^{\prime }$. But the latter means that

(25) $$G\left(q,c,\Lambda \right)\equiv F\left(q,\Lambda \right).$$

We have thus arrived at the same representation as in the previous section, but without assuming context-independent mapping, free choice, or no-fine-tuning.

## 4. Conclusions

We have seen that if one does not make any constraining assumptions about how a measurement outcome in a system depends on settings, local or remote, and if one adopts the CbD definition of generalized (non)contextuality, the derivation of the traditional criteria of (non)contextuality follows with no ontological assumptions, by simply specializing the definition in question to consistently connected systems.

Let us examine possible doubts about the validity of our analysis.

(1) Is not the property of consistent connectedness from which we derive (24) and (25) an assumption? If the distributions of the random variables are only known to us on a sample level, then it is indeed an assumption, as any other statistical hypothesis. However, the discussion in this article deals with the systems known to us precisely, as random variables rather than samples. For example, a standard quantum mechanical computation can yield the precise joint probabilities for the pairs of variables in each context of system $R_{4}$.

(2) By writing $R_{q}^{c}=\gamma \left(q,c,\lambda_{q}^{c}\right)$, have we not made the assumption of “outcome determinism”? The latter means that the values of the relevant arguments uniquely determine the value of $R_{q}^{c}$. What if q,c, and $\lambda_{q}^{c}$ only determine the distribution of the random variable $R_{q}^{c}$ rather than its value? This possibility, however, amounts to introducing yet another random variable among the arguments of the function determining the value of $R_{q}^{c}$:

(26) $$R_{q}^{c}=\gamma \left(q,c,\lambda_{q}^{c},\mu_{q}^{c}\right).$$

This obviously reduces to the initial representation on renaming $\left(\lambda_{q}^{c},\mu_{q}^{c}\right)$ into $\lambda_{q}^{c}$. This observation is, in fact, a short (and generalized) version of a theorem established as early as 1982 by Arthur Fine [12].

(3) Is not the generalized CbD definition of contextuality a form of the no-fine-tuning assumption? This is clearly not true for the original no-fine-tuning assumption [19,20], as the latter is confined to consistently connected systems. However, it has been shown by M. Jones [23] that it is possible to generalize the no-fine-tuning assumption to become a principle that forbids “hidden influences” in ontological models of a system. “Hidden influences” mean dependence of measurement outcomes on factors that do not influence the distributions of these outcomes. Maximizing the probability of $G\left(q,c,\Lambda \right)=G\left(q,c^{\prime },\Lambda \right)$ ensures that the entire difference between the influences of *c* and $c^{\prime }$ on the measurement of *q* is reflected in the difference of their distributions. However, in CbD, this is a consequence of defining (non)contextuality of a system in terms of differences between content-sharing random variables, rather than an assumption about nature or a principle for constructing plausible ontological theories.

(4) Finally, let us address the question about the distinction we make between ontological assumptions and a classificatory definition. Is it defensible? Is not any assumption convertible into a definition and vice versa? Specifically, the traditional analysis of contextuality can be presented as a definition according to which a (consistently connected) system is noncontextual if a representation

(27) $$\left(R_{q}^{c}:q≺c\right)\overset{d}{=}\left(F\left(q,\Lambda \right):q≺c\right)$$

exists, and it is noncontextual otherwise. Not denying this obvious possibility, it is nevertheless reasonable to ask how such a definition is motivated. The assumptions of context-independent mapping, free choice, and no-fine-tuning serve to provide this motivation. (Let us note in passing that most of the traditional analyses of contextuality confuse the distributional Equation (27) with the equality $R_{q}^{c}=F\left(q,\Lambda \right)$. The ensuing logical problems are discussed in [9,21]). In our analysis, we begin with a hidden variable model that cannot be empirically false, $R_{q}^{c}=\gamma \left(q,c,\lambda_{q}^{c}\right)$, and reduce it to $R_{q}^{c}\overset{d}{=}G\left(q,c,\Lambda \right)$, that cannot be false either. Then we pose the question about the difference between random variables $R_{q}^{c}$ and $R_{q}^{c^{\prime }}$, measuring the same content in different contexts. Asked about the motivation for this question, the obvious answer is that we are interested in the dependence of measurements on their contexts. The maximal probability of $S_{q}^{c}=S_{q}^{c^{\prime }}$ in the coupling

(28) $$\left(S_{q}^{c}=F\left(q,c,\Lambda \right),S_{q}^{c^{\prime }}=F\left(q,c^{\prime },\Lambda \right)\right)$$

provides the answer for $R_{q}^{c}$ and $R_{q}^{c^{\prime }}$ taken in isolation from their contexts. Finally, we ask the question if thus measured differences between $R_{q}^{c}$ and $R_{q}^{c^{\prime }}$ are compatible with their respective contexts, by finding out if the maximal probability of $S_{q}^{c}=S_{q}^{c^{\prime }}$ remains the same or decreases within the overall couplings of the system. If asked about the motivation for this question, the answer is that the situations when these maximal probabilities decrease (for some $R_{q}^{c}$ and $R_{q}^{c^{\prime }}$) indicate a special form of dependence of measurements on their contexts, beyond the difference of their distributions. There seems to be no assumptions about nature that would pertain to this distinction (see point 3 above). To summarize, the point we make in this paper is not based on the admittedly blurry distinction between definitions and assumptions. Rather, it is based on dealing with a representation, $\gamma \left(q,c,\lambda_{q}^{c}\right)$ or $G\left(q,c,\Lambda \right)$, that is universally applicable, and does not therefore involve any ontological assumptions.
